# Patients with Active Acromegaly are at High Risk of 25(OH)D Deficiency

**DOI:** 10.3389/fendo.2015.00089

**Published:** 2015-06-02

**Authors:** Jowita Halupczok-Żyła, Aleksandra Jawiarczyk-Przybyłowska, Marek Bolanowski

**Affiliations:** ^1^Endocrinology, Diabetology and Isotope Therapy, Wroclaw Medical University, Wroclaw, Poland

**Keywords:** acromegaly, GH, IGF-1, vitamin D, calcium, phosphate

## Abstract

Acromegaly is a chronic disease characterized by hypersecretion of growth hormone (GH) and insulin-like growth factor 1 (IGF-1). Electrolyte disturbances such as hypercalcemia and hyperphosphatemia are reported in patients with this disorder. There is limited data on vitamin D status in subjects with acromegaly. The aim of the study was to determine calcium, inorganic phosphate, magnesium, alkaline phosphatase, and 25(OH)D levels with regard to the activity of the disease. We also studied correlations of 25(OH)D and IGF-1, GH, body mass, body mass index, and age. A study group consisted of 55 acromegalic patients, and was divided into three subgroups: active acromegaly (AA), well-controlled acromegaly (WCA), cured acromegaly (CA). We enrolled 29 healthy subjects to a control group (CG). Vitamin D deficiency was recorded in all AA patients, 13 WCA patients (92.86%), 10 CA patients (62.5%), and 13 controls (54.17%). The highest 25(OH)D levels were found in the CG group and the lowest in the AA group (*p* = 0.012). The dose of octreotide did not influence serum 25(OH)D levels. A significant positive correlation between IGF-1 and 25(OH)D levels was observed in the AA group (*r* = 0.58, *p* = 0.024). Inorganic phosphate levels were the highest in the AA group. In conclusion, active acromegalic patients have lower 25(OH)D levels in comparison with the CG and are at higher risk of vitamin D deficiency.

## Introduction

Acromegaly is a rare, chronic disease caused by hypersecretion of growth hormone (GH), usually from a pituitary adenoma. Most GH effects are mediated by insulin-like growth factor 1 (IGF-1), which is synthesized mainly in the liver and also in peripheral tissues (1). An association between GH/IGF-1 axis and vitamin D has been studied over past years (1–7). It is thought that vitamin D stimulates production of IGF-1 and IGF binding protein-3 (IGFBP-3) in the liver by direct transcription regulation and/or by enhancing GH stimulation. Furthermore, IGF-1 causes an increase in 1,25-dihydroxyvitamin D [1,25(OH)_2_D] levels by stimulating the expression and activity of the 1a-hydroxylase in the kidney, which leads to an altered calcium–phosphate balance in uncontrolled acromegaly (4, 8).

Exposure to ultraviolet (UV) radiation in sunlight initiates production of previtamin D3 from 7-dehydrocholesterol (provitamin D_3_). Previtamin D_3_ undergoes rapid thermal isomerization to cholecalciferol (vitamin D_3_) (9). After synthesis in the skin (cholecarciferol) or ingestion with the diet (ergocalciferol and cholecalciferol), vitamin D is hydroxylated to 25-hydroxyvitamin D [25(OH)D] in the liver. The total 25-hydroxyvitamin D refers to the sum of vitamin D_2_ and D_3_ 25-hydroxymetabolites. The second hydroxylation takes place mainly in the kidney and 25(OH)D is converted to its biologically active form [1,25(OH)_2_D] (8–10). Other cell types such as keratinocytes, activated macrophages, bone, breast, prostate, and brain cells can also synthesize [1,25(OH)_2_D] (9, 11). Vitamin D receptor (VDR) is expressed in most tissues throughout the body (12). The combined presence of 1a-hydroxylase and VDR in several tissues suggests a paracrine/autocrine role of [1,25(OH)_2_D_3_] (9–11). There is agreement that 25(OH)D is regarded as the best measurement of overall vitamin D (8, 10, 12).

Until recently, vitamin D deficiency was associated mainly with impaired bone mineralization leading to conditions such as rickets in children, osteomalacia, osteoporosis, myopathy, and increased fracture risk (9, 12, 13). It is also reported that fracture risk is high in patients with acromegaly (14). During the last decades, research has brought evidence that vitamin D acts like a pleiotropic hormone (8, 15). Vitamin D deficiency is considered to be a risk factor of cardiovascular disease, cancers, diabetes, respiratory disease, and all-cause mortality (12, 13, 16). Moreover, normal vitamin D status seems to be protective against infectious diseases, autoimmune diseases, neurocognitive dysfunction and mental illness, infertility, adverse pregnancy, and birth outcomes (16).

The aim of our study was to determine vitamin D status and calcium/phosphate homeostasis in patients with acromegaly with regard to the activity of the disease and to evaluate the association between vitamin D and GH, IGF-1, body mass, BMI, and age.

## Materials and Methods

We enrolled 55 patients with acromegaly aged 23–83 years (16 males and 39 females; mean age 50.85 ± 5.24 years). Based on clinical findings and hormonal evaluation (GH, IGF-1), we divided the study group into three subgroups: (1) active acromegaly group (AA), 20 patients; (2) well-controlled acromegaly group (WCA), 17 patients; (3) cured acromegaly (CA), 18 patients. Normal IGF-1 values (sex- and age-matched) and GH levels during oral glucose tolerance test (OGTT) lower than 1.0 μg/l (ng/ml) were the criteria used for classifying patients as cured or well-controlled. Patients who had undergone successful surgical treatment were assigned to the CA group. Subjects, after failed surgery, receiving treatment with 10–30 mg of long-acting octreotide (Sandostatin LAR, Novartis) administered intramuscularly every 28 days were recruited to the WCA group. Patients who did not meet criteria for cured or WCA were included in the AA group. We selected a control group (CG) of 29 patients aged 21–77 years (6 males and 23 females) by matching subjects and controls by sex and age. The CG showed no clinical symptoms of acromegaly and had normal IGF-1 (age- and sex-matched) and GH values. All subjects in AA and WCA groups were treated with long-acting octreotide. In the AA group, 2 subjects received 10 mg dose, 5 subjects 20 mg dose and 13 subjects 30 mg dose. Not all of the AA patients received the dose of 30 mg due to drug intolerance. In the WCA group, 6 patients received 20 mg dose and 11 patients 30 mg dose. Among the study group, 23 patients required substitution therapy with hydrocortisone and 22 with l-thyroxine. There was no statistically significant difference between the numbers of patients requiring hormonal replacement therapy in each subgroup. Diabetes mellitus treated with oral hypoglycemic agents was observed in 13 study group patients. None of the patients received calcium or vitamin D supplementation. All participants had normal renal and hepatic function. For the purposes of statistical analysis, we created three classifications. The first classification was based on the degree of disease activity (AA; WCA; CA; CG). The second classification was used to analyze the differences between the AA group, patients with cured and WCA (WCA + CA) and controls (CG). The third classification compared the study group (AA + WCA + CA) and the CG. The study was approved by ethics committee of Wroclaw Medical University. All patients were recruited from the Department of Endocrinology, Diabetology and Isotope Therapy, Wroclaw Medical University. Written informed consent was obtained from all patients.

We recorded patients’ weight (kg) and height (m) and calculated their body mass index (BMI). Fasting venous blood samples were collected from all participants. We evaluated adrenal, somatotropic, thyroid, and gonadal axes, and prolactin (PRL). The adrenocorticotropic hormone (ACTH), cortisol, GH, thyrotropic hormone (TSH), free triiodothyronine (fT3) and free thyroxine (fT4), luteinizing hormone (LH), follicle-stimulating hormone (FSH), estradiol (E2), total testosterone (T), and PRL were measured by chemiluminescence immunoassay method using Immulite 2000 kits (DPC, Germany or USA; Siemens, USA). Reference ranges were as follows: ACTH 0–46 pg/ml; GH 0.06–5 μg/l (ng/ml); TSH 0.4–4.0 mIU/l; fT_3_ 2,76–6,45 pmol/l; fT_4_ 11.5–22.7 pmol/l; LH postmenopausal women 11.3–39.8 mIU/ml, women in follicular phase 1.1–11.6 mIU/ml, men 0.8–7.6 mIU/ml; FSH postmenopausal women 9.7–111 mIU/ml, women in follicular phase 2.8–11.3 mIU/ml, men 0.7–11.1 mUI/ml; E_2_ postmenopausal women 30–140 pg/ml, women in follicular phase 0–160 pg/ml; T women 0.2–0.8 ng/ml, men 1.29–7.67 ng/ml, PRL women 1.9–25 ng/ml, men 2.5–17 ng/ml. Cortisol levels were measured by chemiluminescent microparticle immunoassay using Architect i1000SR (Abbott Laboratories, Abbott Park, IL, USA). Reference ranges: before 10 a.m. 3.7–19.4 μg/dl; after 5 p.m. 2.9–17.3 μg/dl. Urine free cortisol excretion was measured using a radioimmunoassay method (Immunotech, Beckman Coulter Inc., Prague, Czech Republic), reference range: 14.0–75.0 μg/24 h.

Serum IGF-1 levels were assessed by radioimmunologic assay using IGF-1-D-RIT-CT kit (BioSource Europe S.A., Nivelles, Belgium); reference ranges were sex- and age-matched. IGF-1 levels were expressed in relation to the upper limit of normal (ULN) for age (patient’s IGF-1 concentration divided by IGF-1 upper reference range limit matched for age). In the study group, we assessed plasma GH concentrations at 0, 60, and 120 min after oral administration of 75 g glucose. Serum 25(OH)D levels were measured in 45 study group patients (15 AA, 14 WCA, and 16 CA subjects) and 24 controls using radioimmunoassay kits (DIAsource, ImmunoAssays S.A., Louvain-la-Neuve, Belgium), reference range: 20–70 ng/ml (summer), 10–60 ng/ml (winter). The intra- and inter-assay coefficients of variation (CVs) were 7.3 and 7.2%, respectively. Seasonality was based on the date of blood sample collection. October–April period corresponded to winter season and May–September period represented summer season. We used following ranges of 25(OH)D concentrations indicating vitamin D deficiency [<20 ng/ml (<50 nmol/l)], suboptimal status [20–30 ng/ml (50–75 nmol/l)], optimal status [30–50 ng/ml (75–125 nmol/l)] (16). Serum calcium, inorganic phosphate, magnesium, and alkaline phosphatase were measured using colorimetric assays on an Architect c4000 (Abbott Laboratories, Abbott Park, IL, USA). Reference ranges were as follows: calcium 4.2–5.15 mEq/l; inorganic phosphate 2.7–4.5 mg/dl; magnesium 1.6–2.6 mg/dl; alkaline phosphatase 40–150 IU/l. Statistical analysis was performed using Statistica software for Windows, version 7.0 (StatSoft, Tulsa, Oklahoma, USA). The mean, 95% confidence interval, standard deviation, and median were determined for all variables. The Shapiro–Wilk’s test was used to test the normality of the variables. Student’s *t* test was applied when normality was indicated. A Mann–Whitney *U* test was used for data not normally distributed. Correlations were determined using Pearson’s test or Spearman’s rank correlation test as appropriate based on variable distribution. *p* values <0.05 were considered statistically significant.

## Results

The highest mean body mass was observed in the AA group and the lowest in the CG group. We found statistically significant differences between groups: AA and CG (*p* = 0.014); CA and CG (*p* = 0.005); WCA + CA and CG (*p* = 0.009); AA + WCA + CA and CG (*p* = 0.004). The CA group had the highest mean BMI, while the CG had the lowest. The comparison between the CG group and WCA, CA, WCA + CA and AA + WCA + CA groups revealed statistically significant differences (*p* < 0.003, *p* = 0.001, *p* = 0.001, *p* = 0.003; respectively) (Table 1).

**Table 1:** **Demographic characteristics of the study and control groups**.

Group	Sex (*n*)		Age (years)	Body mass (kg)	Height (m)	BMI (kg/m^2^)
	Female	Male	
AA	10	10	50.85 ± 15.24	88.10 ± 21.89^a^	1.73 ± 0.11^b^^,^^c^^,^^d^	29.31 ± 5.18
WCA	3	14	55.35 ± 12.45	81.47 ± 14.44	1.65 ± 0.09	29.73 ± 4.73^a^
CA	15	3	54.00 ± 11.52	88.00 ± 14.59^a^	1.66 ± 0.10	31.71 ± 4.48^a^
CG	23	6	47.86 ± 15.70	74.78 ± 14.90	1.67 ± 0.09	26.83 ± 4.55
WCA + CA	18	17	54.66 ± 11.83	84.83 ± 14.68^a^	1.66 ± 0.09	30.75 ± 4.65^a^
AA + WCA + CA	28	27	53.27 ± 13.16	86.02 ± 17.52^a^	1.68 ± 0.11	30.23 ± 4.85^a^

*AA, active acromegaly; WCA, well-controlled acromegaly; CA, cured acromegaly; CG, control group*.

*^a^*p* < 0.05 compared to CG*.

*^b^*p* < 0.05 compared to WCA*.

*^c^*p* < 0.05 compared to CA*.

*^d^*p* < 0.05 compared to WCA + CA*.

Vitamin D deficiency occurred in all AA patients, 13 WCA patients (92.86%), 10 CA patients (62.5%) and 13 controls (54.17%). Suboptimal levels of vitamin D were found in one WCA patient (7.14%), six CA patients (37.5%), and nine CG patients (37.5%). Only two CG patients (8.33%) showed optimal vitamin D concentrations.

**Figure 1:**
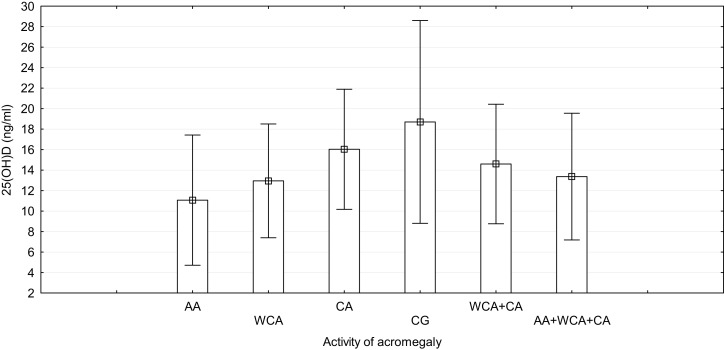
**Concentration of 25(OH)D with regard to the activity of acromegaly**. AA, active acromegaly; WCA, well-controlled acromegaly; CA, cured acromegaly; CG, control group.

The highest 25(OH)D levels were found in the CG group and the lowest in the AA group (the difference was statistically significant, *p* = 0.012) (Figure 1). In the WCA and CA groups, 25(OH)D concentrations were also lower compared to the CG group; however, the differences were not statistically significant (*p* = 0.109, *p* = 0.473; respectively). The differences in 25(OH)D levels among the AA, WCA, and CA groups were not statistically significant, although the highest levels of 25(OH)D were noted in the CA group. Based on the third classification (AA + WCA + CA vs. CG), higher 25(OH)D concentrations were found in CG group, the difference was statistically significant (*p* = 0.047). Other comparisons (AA and WCA + CA, WCA + CA and CG) did not reveal statistically significant differences (Table 2). We found no seasonal variations in 25(OH)D levels in the AA, AA + WCA + CA and CG groups. There was no relationship between the dose of octreotide and serum 25(OH)D levels.

**Table 2:** **Laboratory tests results in the study and control groups**.

Group	GH 0 min (ng/ml)	GH 60 min (ng/ml)	GH 120 min (ng/ml)	IGF-1 (ng/ml)	25(OH)D (ng/ml)	Calcium (mEq/l)	Inorganic phosphate (mg/dl)	Magnesium (mg/dl)	Alkaline phosphatase (U/l)
AA	11.56 ± 14.93^a^^,^^b^^,^^c^^,^^d^	9.17 ± 10.57^a^^,^^b^^,^^c^^,^^d^	9.65 ± 10.56^a^^,^^b^^,^^c^^,^^d^	540.50 ± 263.97^a^^,^^b^^,^^c^^,^^d^	11.06 ± 6.58^a^	4.88 ± 0.22	3.87 ± 0.65^a^^,^^b^^,^^c^^,^^d^	1.99 ± 0.25	129.17 ± 59.92
WCA	2.21 ± 2.11^a^^,^^c^	1.42 ± 1.68^c^	1.44 ± 1.50^a^^,^^c^	216.57 ± 129.71	12.95 ± 5.55	4.69 ± 0.72	3.36 ± 0.47	1.98 ± 0.40	113.40 ± 91.13
CA	1.32 ± 2.04	0.31 ± 0.23	0.98 ± 2.79	164.92 ± 86.19	16.03 ± 5.86	4.80 ± 0.29	3.43 ± 0.39	1.93 ± 0.37	89.29 ± 22.32
CG	1.51 ± 2.82	2.39 ± 5.76	0.50 ± 1.09	153.94 ± 79.65	18.70 ± 9.90	4.78 ± 0.29	3.37 ± 0.57	2.04 ± 0.28	143.47 ± 158.26
WCA + CA	1.75 ± 2.09^a^	0.85 ± 1.29^a^	1.20 ± 2.24^a^	189.99 ± 110.98	14.59 ± 5.83	4.75 ± 0.53	3.40 ± 0.42	1.95 ± 0.37	103.47 ± 70.77
AA + WCA + CA	5.32 ± 10.19^a^	3.77 ± 7.42	4.27 ± 7.70^a^	317.45 ± 247.44^a^	13.42 ± 6.25^a^	4.80 ± 0.44	3.59 ± 0.57	1.96 ± 0.32	112.86 ± 66.35

*AA, active acromegaly; WCA, well-controlled acromegaly; CA, cured acromegaly; CG, control group*.

*^a^*p* < 0.05 compared to CG*.

*^b^*p* < 0.05 compared to WCA*.

*^c^*p* < 0.05 compared to CA*.

*^d^*p* < 0.05 compared to WCA + CA*.

**Figure 2:**
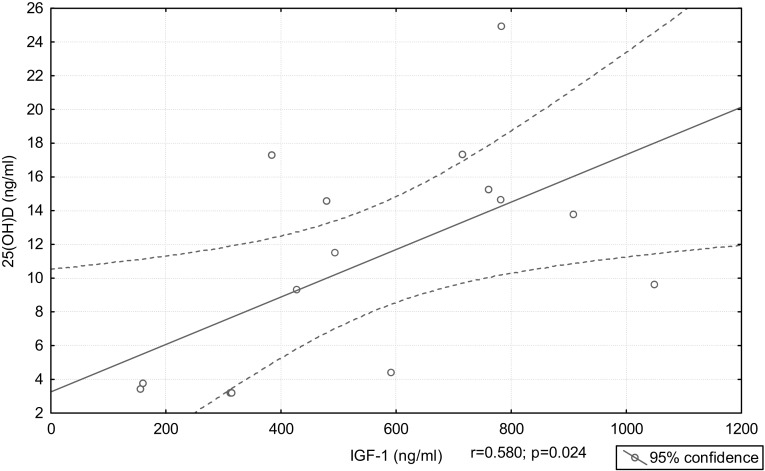
**Correlation between 25(OH)D and IGF-1 in the active acromegaly group**.

A significant positive correlation between IGF-1 levels and 25(OH)D levels was noted only in the AA group (*r* = 0.580, *p* = 0.024) (Figure 2). There was no correlation between the GH levels at 0, 60, and 120 min during OGTT and 25(OH)D levels. We found negative correlation between age and 25(OH)D levels in the CA, WCA + CA and AA + WCA + CA groups (*r* = −0.686, *p* = 0.05; *r* = −0.494, *p* = 0.006; *r* = −0.421, *p* = 0.004; respectively). There was no correlation between body mass, BMI, and 25(OH)D levels.

We also investigated correlations between 25(OH)D and calcium, inorganic phosphate, magnesium concentrations. There were significant positive correlations between calcium levels and 25(OH)D levels in the WCA and WCA + CA groups (*r* = 0.697, *p* = 0.025; *r* = 0.442, *p* = 0.039; respectively). Magnesium levels correlated positively with 25(OH)D levels in the WCA group (*r* = 0.847, *p* = 0.016).

**Figure 3:**
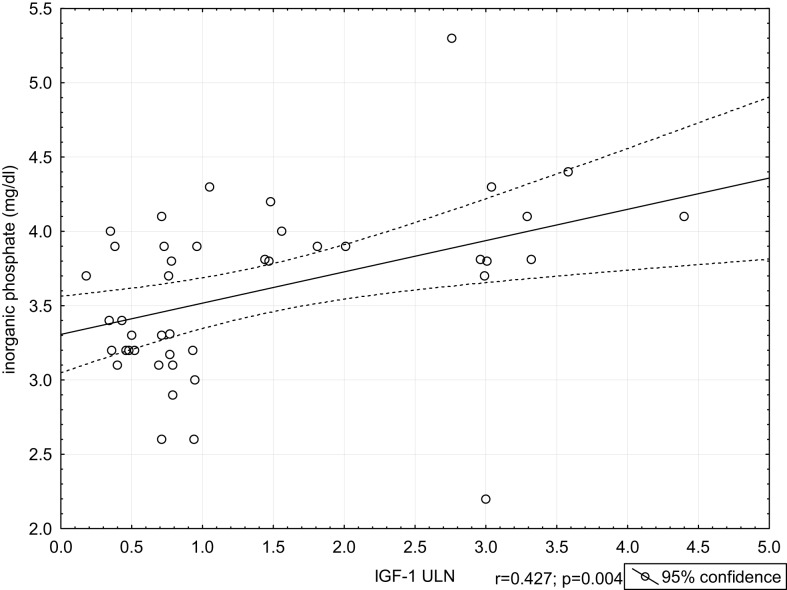
**Correlation between inorganic phosphate and IGF-1 ULN in the AA + WCA + CA group**.

Calcium concentrations were the highest in the AA group; however, the differences were not statistically significant, as compared to other groups. We did not find any significant differences in magnesium levels among groups. Plasma levels of inorganic phosphate were the highest in the AA group. We noted statistically significant differences in inorganic phosphate levels between groups: AA and CG (*p* = 0.012), AA and WCA (*p* = 0.029), AA and CA (*p* = 0.015), AA and WCA + CA (*p* = 0.006). We found significant positive correlation between inorganic phosphate and IGF-1 in the AA group (*r* = 0.582, *p* = 0.014) and between inorganic phosphate and IGF-1 ULN in the AA + WCA + CA group (*r* = 0.427; *p* = 0.004) (Figure 3). Inorganic phosphate levels correlated positively with GH concentrations at 0 min in the CG group (*r* = 0.516, *p* = 0.028). There were no correlations between calcium levels, alkaline phosphatase levels and IGF-1 levels in the AA group. We found negative correlation between calcium levels and GH levels at 0 min in the CA group (*r* = −0.641, *p* = 0.014). PRL concentrations were higher in the AA, WCA, and CA groups compared to controls. There were no significant differences in the levels of other hormones (data not shown).

## Discussion

We have not found any data on the prevalence of vitamin D deficiency in patients with acromegaly. Vitamin D deficiency and insufficiency are global health problem; however, optimal ranges of vitamin D are still not clearly defined (10, 16). In our study, all patients with AA demonstrated 25(OH)D deficiency, whereas in the CG only 13 patients (54.17%). This may suggest that patients with uncontrolled acromegaly are more prone to develop 25(OH)D deficiency. Classification of vitamin D status we used is similar to the Endocrine Society Clinical Practice Guidelines where vitamin D deficiency is defined as a 25(OH)D below 20 ng/ml and insufficiency as a 25(OH)D of 21–29 ng/ml (17, 18). Epidemiological data on vitamin D status in Poland is limited. In a recent, large cross-sectional study, 67.5% of the Polish adult population had 25(OH)D level <20 ng/ml, 22.8% had suboptimal level of 20–30 ng/ml and only 8.4% had optimal 25(OH)D level (30–50 ng/ml) (19). Hypovitaminosis D is associated with inadequate sunlight exposure, obesity, dark skin pigmentation, fat malabsorption, nephrotic syndrome, renal failure, hepatic failure, and medications (anticolvulsants, glucocorticoids, AIDS/HIV drugs) (12, 20). 25(OH)D levels were lower in patients with AA compared to the CG. This may be related to the higher body mass in AA group. Płudowski et al. showed that high body mass and high BMI are risk factors of vitamin D deficiency. They found significantly lower 25(OH)D concentrations in patients with obesity compared to patients with BMI <30 kg/m^2^ (19). It is well-established that vitamin D deficiency is associated with obesity. Vitamin D is fat-soluble and stored in adipose tissue. Additionally, obese patients have less exposure to sunlight because of lower exercise and mobility (15, 21). Negative correlations between 25(OH)D and BMI, body mass were previously reported (19). Although we are not able to confirm this fact, 25(OH)D levels decrease with age due to less time spent outdoors and decreased vitamin D production efficiency (22). We also demonstrated negative correlation between age and 25(OH) levels (CA and WCA + CA groups). In other study, the highest 25(OH)D concentrations were reported in elderly patients (>70 years), while the lowest in the patients aged 15–25 years. This might be explained by the fact that elderly people often take vitamin D supplements (19).

Long-term and short-term treatment with somatostatin analogs in patients with acromegaly does not affect vitamin D levels (23, 24). Somatostatin analogs cause impairment of fat absorption which can lead to loss of fat-soluble vitamins (25). This might also influence vitamin D status in patients with active and WCA (AA and WCA groups). In our study, we found no relationship between the dose of octreotide and 25(OH)D concentrations.

Vitamin D and IGF-1 both have a broad spectrum of effects and their interrelations are extremely complex (8). We showed a positive correlation between IGF-1 and 25(OH)D levels in AA patients. Several studies disclosed a positive correlation between IGF-1 and 25(OH)D concentrations (7, 26–28). Hyppönen et al. reported a positive association between 25(OH)D and IGF-1, with a linear increase in IGF-1 until 25(OH)D concentrations reached 75–85 nmol/l, after which this effect reached a plateau (7). IGF-1 and 25(OH)D might up-regulate each other. 25(OH)D can increase IGF-1 receptor expression, thus promoting IGF-1 action (28). Furthermore, an *in vitro* study on cultured human fetal epiphyseal chondrocytes showed that vitamin D stimulates expression of several GH–IGF axis genes such as IGF-1, IGFBP-3, and growth hormone receptor (GHR) (3). On the other hand, IGF-1 increases vitamin D activity by stimulation of renal 1a-hydroxylase (28). Although 25(OH)D levels were lower in the AA group, suggesting high IGF-1 seems to be associated with low 25(OH)D levels, IGF-1 was positively correlated with 25(OH)D in the AA group. These findings point out that the association between IGF-1 and vitamin D status is more complex.

In our study, we did not find correlations between 25(OH)D levels and GH levels at 0, 60, and 120 min of the OGTT. It is not clear if GH actions on 1,25(OH)2D renal production are mediated by IGF-1 (6). In patients with AA, GH receptor antagonist (pegvisomant) administration resulted in normalization of IGF-1 and 1,25(OH)_2_D decrease. Interestingly, 25(OH)D concentrations remained unaffected (29). GH replacement in healthy adults increased 1,25(OH)_2_D levels (6, 30). In another study, patients with acromegaly had significantly increased 1,25(OH)_2_D concentrations, however there was no correlation between GH and 1,25(OH)_2_D levels. This suggests that other factors may be responsible for elevated 1,25(OH)_2_D (2).

We demonstrated a positive correlation between 25(OH)D levels and calcium levels (in WCA and CA + WCA groups). 1,25(OH)_2_D regulates bone metabolism and stimulates intestinal calcium and phosphate absorption and renal calcium reabsorption. Moreover, 1,25(OH)2D inhibits parathormone (PTH) release from the parathyroid glands. Renal production of 1,25(OH)2D is therefore regulated by phosphate, PTH, and fibroblast growth factor 23 (FGF-23) (8).

Hypercalcemia is rarely encountered in patients with acromegaly and might be PTH-dependent or 1,25(OH)_2_D-dependent (1, 31). GH excess is also associated with hypercalciuria and nephrolithiasis (31, 32). Kamenický et al. found that increased production of calcitriol in acromegaly is responsible for hypercalciuria and increased fasting plasma calcium concentrations (33). We did not observe any significant differences in calcium levels among the groups. Hyperphosphatemia is a common finding in patients with acromegaly (6). The highest inorganic phosphate levels were observed in patients with AA (AA group). We also found positive correlation between IGF-1 and phosphate concentrations in the AA group. Bianda et al. reported that renal phosphate reabsorption increases in response to GH treatment, whereas this effect is not present during IGF-1 therapy. A direct effect of GH on tubular transport of phosphate, stimulation of local production of IGF-1 acting in a paracrine way or GH effects via other local or systemic regulators might be the mechanisms explaining this fact (6).

The limitation of our study is that we did not measure levels of 1,25(OH)2D, PTH, and FGF-23, which have a strong influence on calcium/phosphate status.

In conclusion, patients with AA have lower 25(OH)D concentrations compared to controls and are at higher risk of vitamin D deficiency. In AA IGF-1 concentrations correlate positively with 25(OH)D levels. Patients with AA present a tendency to higher inorganic phosphate levels than healthy subjects. Further research is needed to fully understand the complex interactions GH/IGF-1 axis and vitamin D.

## Conflict of Interest Statement

The authors declare that the research was conducted in the absence of any commercial or financial relationships that could be construed as a potential conflict of interest.

## References

[1] Anthony JR Ioachimescu AG. Acromegaly and bone disease. Curr Opin Endocrinol Diabetes Obes (2014) 21:476–82. 10.1097/MED.0000000000000109 25354045

[2] Brown DJ Spanos E MacIntyre I. Role of pituitary hormones in regulating renal vitamin D metabolism in man. Br Med J (1980) 280:277–8. 10.1136/bmj.280.6210.277 7357340 PMC1600180

[3] Fernández-Cancio M Audi L Carrascosa A Toran N Andaluz P Esteban C Vitamin D and growth hormone regulate growth hormone/insulin-like growth factor (GH-IGF) axis gene expression in human fetal epiphyseal chondrocytes. Growth Horm IGF Res (2009) 19:232–7. 10.1016/j.ghir.2008.10.004 19056306

[4] Ameri P Giusti A Boschetti M Bovio M Teti C Leoncini G Vitamin D increases circulating IGF-1 in adults: potential implication for the treatment of GH deficiency. Eur J Endocrinol (2013) 169:767–72. 10.1530/EJE-13-0510 24005315

[5] Wright NM Papadea N Wentz B Hollis B Willi S Bell NH Increased serum 1,25-dihydroxyvitamin D after growth hormone administration is not parathyroid hormone-mediated. Calcif Tissue Int (1997) 61:101–3. 10.1007/s002239900303 9312396

[6] Bianda T Hussain MA Glatz Y Bouillon R Froesch ER Schmid C. Effects of short-term insulin-like growth factor-I or growth hormone treatment on bone turnover, renal phosphate reabsorption and 1,25 dihydroxyvitamin D3 production in healthy man. J Intern Med (1997) 241:143–50. 10.1046/j.1365-2796.1997.94101000.x 9077371

[7] Hyppönen E Boucher BJ Berry DJ Power C. 25-hydroxyvitamin D, IGF-1, and metabolic syndrome at 45 years of age: a cross-sectional study in the 1958 British Birth Cohort. Diabetes (2008) 57:298–305. 10.2337/db07-1122 18003755

[8] Ameri P Giusti A Boschetti M Murialdo G Minuto F Ferone D. Interactions between vitamin D and IGF-I: from physiology to clinical practice. Clin Endocrinol (Oxf) (2013) 79:457–63. 10.1111/cen.12268 23789983

[9] Holick MF The vitamin D deficiency pandemic: a forgotten hormone important for health. Public Health Rev (2010) 32:267–83.

[10] Peterson CA Tosh AK Belenchia AM. Vitamin D insufficiency and insulin resistance in obese adolescents. Ther Adv Endocrinol Metab (2014) 5:166–89. 10.1177/2042018814547205 25489472 PMC4257980

[11] Verstuyf A Carmeliet G Bouillon R Mathieu C. Vitamin D: a pleiotropic hormone. Kidney Int (2010) 78:140–5. 10.1038/ki.2010.17 20182414

[12] Dobnig H. A review of the health consequences of the vitamin D deficiency pandemic. J Neurol Sci (2011) 311:15–8. 10.1016/j.jns.2011.08.046 21939984

[13] Khaw K-T Luben R Wareham N. Serum 25-hydroxyvitamin D, mortality, and incident cardiovascular disease, respiratory disease, cancers, and fractures: a 13-y prospective population study. Am J Clin Nutr (2014) 100:1361–70. 10.3945/ajcn.114.086413 25332334 PMC4196486

[14] Mazziotti G Biagioli E Maffezzoni F Spinello M Serra V Maroldi R Bone turnover, bone mineral density, and fracture risk in acromegaly: a meta-analysis. J Clin Endocrinol Metab (2015) 100(2):384–94. 10.1210/jc.2014-2937 25365312

[15] Rosen CJ Adams JS Bikle DD Black DM Demay MB Manson JE The nonskeletal effects of vitamin D: an Endocrine Society scientific statement. Endocr Rev (2012) 33:456–92. 10.1210/er.2012-1000 22596255 PMC3365859

[16] Pludowski P Holick MF Pilz S Wagner CL Hollis BW Grant WB Vitamin D effects on musculoskeletal health, immunity, autoimmunity, cardiovascular disease, cancer, fertility, pregnancy, dementia and mortality-a review of recent evidence. Autoimmun Rev (2013) 12:976–89. 10.1016/j.autrev.2013.02.004 23542507

[17] Płudowski P Karczmarewicz E Bayer M Carter G Chlebna-Sokół D Czech-Kowalska J Practical guidelines for the supplementation of vitamin D and the treatment of deficits in Central Europe - recommended vitamin D intakes in the general population and groups at risk of vitamin D deficiency. Endokrynol Pol (2013) 64:319–27. 10.5603/EP.2013.0012 24002961

[18] Holick MF Binkley NC Bischoff-Ferrari HA Gordon CM Hanley DA Heaney RP Evaluation, treatment, and prevention of vitamin D deficiency: an Endocrine Society clinical practice guideline. J Clin Endocrinol Metab (2011) 96:1911–30. 10.1210/jc.2011-0385 21646368

[19] Płudowski P Konstantynowicz J Jaworski M Abramowicz P Ducki C. Assessment of vitamin D status in Polish adult population. Stand Med Pediatr (2014) 11:609–17. 10.5603/EP.2014.0015 24802733

[20] Holick MF Vitamin D and health: evolution, biologic functions, and recommended dietary intakes for vitamin D. Clin Rev Bone Miner Metab (2009) 7:2–19. 10.1007/s12018-009-9026-x

[21] Muscogiuri G Sorice GP Ajjan R Mezza T Pilz S Prioletta A Can vitamin D deficiency cause diabetes and cardiovascular diseases? Present evidence and future perspectives. Nutr Metab Cardiovasc Dis (2012) 22:81–7. 10.1016/j.numecd.2011.11.001 22265795

[22] Pludowski P Grant WB Bhattoa HP Bayer M Povoroznyuk V Rudenka E Vitamin D status in central Europe. Int J Endocrinol (2014) 2014:589587. 10.1155/2014/589587 24790600 PMC3984788

[23] Cappelli C Gandossi E Agosti B Cerudelli B Cumetti D Castellano M Long-term treatment of acromegaly with lanreotide: evidence of increased serum parathormone concentration. Endocr J (2004) 51:517–20. 10.1507/endocrj.51.517 15644568

[24] Ajmal A Haghshenas A Attarian S Barake M Tritos NA Klibanski A The effect of somatostatin analogs on vitamin D and calcium concentrations in patients with acromegaly. Pituitary (2014) 17:366–73. 10.1007/s11102-013-0514-0 24002366

[25] Fiebrich H-B Van Den Berg G Kema IP Links TP Kleibeuker JH Van Beek AP Deficiencies in fat-soluble vitamins in long-term users of somatostatin analogue. Aliment Pharmacol Ther (2010) 32:1398–404. 10.1111/j.1365-2036.2010.04479.x 21050243

[26] Gómez JM Maravall FJ Gómez N Navarro MA Casamitjana R Soler J. Relationship between 25-(OH) D3, the IGF-I system, leptin, anthropometric and body composition variables in a healthy, randomly selected population. Horm Metab Res (2004) 36:48–53. 10.1055/s-2004-814103 14983407

[27] Forouhi NG Luan J Cooper A Boucher BJ Wareham NJ. Baseline serum 25-hydroxy vitamin d is predictive of future glycemic status and insulin resistance: the Medical Research Council Ely Prospective Study 1990-2000. Diabetes (2008) 57:2619–25. 10.2337/db08-0593 18591391 PMC2551670

[28] Bogazzi F Rossi G Lombardi M Tomisti L Sardella C Manetti L Vitamin D status may contribute to serum insulin-like growth factor I concentrations in healthy subjects. J Endocrinol Invest (2011) 34:e200–3. 10.3275/7228 20671418

[29] Parkinson C Kassem M Heickendorff L Flyvbjerg A Trainer PJ. Pegvisomant-induced serum insulin-like growth factor-I normalization in patients with acromegaly returns elevated markers of bone turnover to normal. J Clin Endocrinol Metab (2003) 88:5650–5. 10.1210/jc.2003-030772 14671148

[30] Marcus R Butterfield G Holloway L Gilliland L Baylink DJ Hintz RL Effects of short term administration of recombinant human growth hormone to elderly people. J Clin Endocrinol Metab (1990) 70:519–27. 10.1210/jcem-70-2-519 2298863

[31] Shah R Licata A Oyesiku NM Ioachimescu AG. Acromegaly as a cause of 1,25-dihydroxyvitamin D-dependent hypercalcemia: case reports and review of the literature. Pituitary (2012) 15(Suppl 1):S17–22. 10.1007/s11102-010-0286-8 21188640

[32] Tsuchiya H Onishi T Takamoto S Morimoto S Fukuo K Imanaka S An acromegalic patient with recurrent urolithiasis. Endocrinol Jpn (1985) 32:851–61. 10.1507/endocrj1954.32.851 3841720

[33] Kamenický P Blanchard A Gauci C Salenave S Letierce A Lombès M Pathophysiology of renal calcium handling in acromegaly: what lies behind hypercalciuria? J Clin Endocrinol Metab (2012) 97:2124–33. 10.1210/jc.2011-3188 22496496

